# Multivariate assessment and attribution of droughts in Central Asia

**DOI:** 10.1038/s41598-017-01473-1

**Published:** 2017-05-02

**Authors:** Zhi Li, Yaning Chen, Gonghuan Fang, Yupeng Li

**Affiliations:** 1 State Key Laboratory of Desert and Oasis Ecology, Xinjiang Institute of Ecology and Geography, Chinese Academy of Sciences, Urumqi, 830011 China; 2 University of Chinese Academy of Sciences, Beijing, 100049 China

## Abstract

While the method for estimating the Palmer Drought Severity Index (PDSI) is now more closely aligned to key water balance components, a comprehensive assessment for measuring long-term droughts that recognizes meteorological, agro-ecological and hydrological perspectives and their attributions is still lacking. Based on physical approaches linked to potential evapotranspiration (PET), the PDSI in 1965–2014 showed a mixture of drying (42% of the land area) and wetting (58% of the land area) that combined to give a slightly wetting trend (0.0036 per year). Despite the smaller overall trend, there is a switch to a drying trend over the past decade (−0.023 per year). We designed numerical experiments and found that PDSI trend responding to the dramatic increase in air temperature and slight change in precipitation. The variabilities of meteorological and agro-ecological droughts were broadly comparable to various PDSI drought index. Interestingly, the hydrological drought was not completely comparable to the PDSI, which indicates that runoff in arid and semi-arid regions was not generated primarily from precipitation. Instead, fraction of glacierized areas in catchments caused large variations in the observed runoff changes.

## Introduction

How will regional droughts change as a consequence of ongoing global warming? Previous assessments of historical changes in drought indices over the past half-century indicate that drought is expected to increase in frequency and severity as a consequence of increasing evaporation driven by global warming and decreased regional precipitation^1–4^. The conclusion indicated that “warmer is more arid”^5, 6^. However, the calculations of drought indices, such as the widely used PDSI by different approaches lead to controversial results in assessing droughts, and parallel researches have highlighted the flaws of approaches for estimating drought index^7–11^. Several recently published studies have produced seemingly conflicting results of how drought is changing under climate change, suggesting that simplified precipitation and temperature changes that rely on poorly parameterized PET, as proposed by Thornthwaite^12^, may give biased results in the context of climate change. For instance, while drought changes over land have not followed a simple intensification of existing patterns, they show little overall change in terms of global averages^13, 14^. These contradictory results draw attention to potential flaws in the underlying methodologies involved.

Furthermore, even though the method for estimating the PDSI using a physical-based approach has now been brought closer to key water balance components, there remains a lack of 1) precipitation with meteorological drought, 2) soil moisture deficits with agro-ecological drought, and 3) runoff with hydrological drought. These differences emphasize the roles of precipitation, evapotranspiration and runoff in climate-induced drought. Hence, a comprehensive discussion is still lacking on the combined topics of drought, drought drivers and drought indices.

Although by no means universal, near-surface air temperature is commonly reported to be higher in recent years, especially in arid/semi-arid regions^15^. Climate change is adding heat to the climate system and much of that heat on land goes into drying. Has large-scale drought been increasing under climate change? The occurrence of disastrous droughts has received enormous and increasing public attention and discussion^16–20^. The most widely accepted interpretation of the underlying cause of drought is that high temperature increases evapotranspiration, thereby drying the soil^21, 22^. However, various drought indices and metrics that comprehensively assess and quantitatively interpret recent changes in droughts have not been adequately factored into the equation.

Drought and water scarcity are key words for regional management in water-stressed regions^23^. Because most dryland soil is relatively infertile and the vegetation cover is sparse, dryland ecosystems are substantially more fragile. Moreover, as climate model projections suggest that increasing greenhouse gas concentrations will lead to increased across many areas in low and mid-latitudes^24, 25^, we should pay closer attention to dryland areas.

Central Asia, which consists of Kazakhstan, Kyrgyzstan, Tajikistan, Turkmenistan, Uzbekistan and the arid region of Northwest China, is located in the hinterland of the Eurasian continent. It is one of the most arid areas in the world, with a unique landscape that features expansive but fragile mountain-oasis-desert ecosystems. The temperature in Central Asia experienced a sharp increase in 1997, and since then has been in a state of high volatility, making the last decade the warmest period on record^26, 27^.

Given this volatility, what are expectations for changes in drought, and what are the potential attributions of drought? Obtaining accurate and quantitative attributions of the causes of drought requires accounting for natural variables based on the calculated model. To this end, we propose conducting a comprehensive assessment for measuring long-term drought that recognizes meteorological, hydrological and agro-ecological perspectives in Central Asia, while also conducting a series of numerical experiments to quantify the sensitivity of the physical-based drought index.

## Results

### Little change in drought based on PDSI

The PDSI is used extensively for operational monitoring and increasingly in studies of climate change. Being closer to standard agro-ecological and hydrological drought indices, the PDSI applies physical-based approaches with potential evaporation calculated as a function of radiation, temperature, humidity, and wind speed^28^. Therefore, we used the most obvious candidate (FAO-endorsed Penman-Monteith [P-M] parameterization) for the calculation of PET^29^, because the P-M is a more accurate, comprehensive and physical-based model of PET.

**Figure 1:**
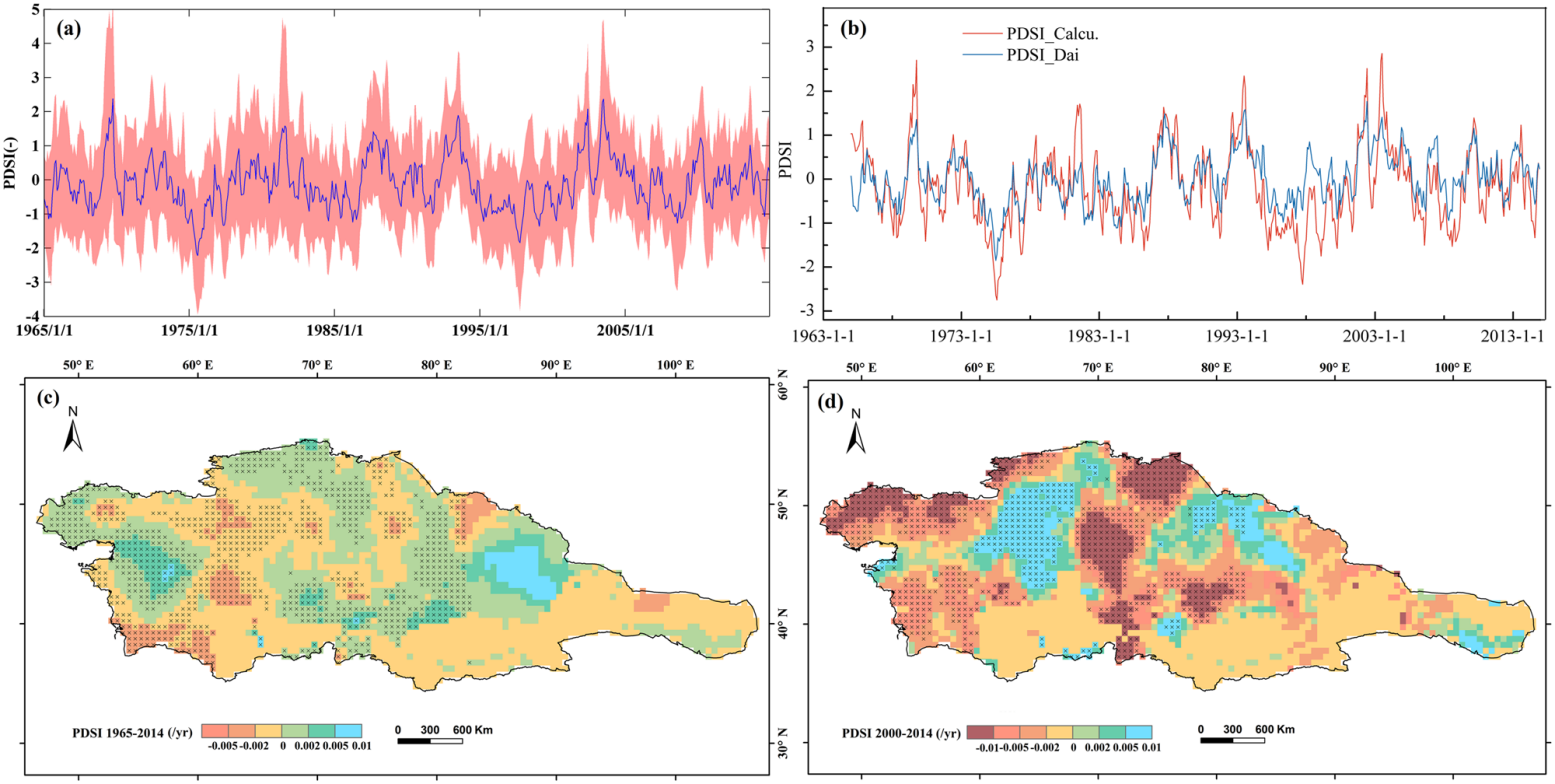
Spatio-temporal changes in the recent droughts over Central Asia during 1965–2014. (**a**) time series of the PDSI; (**b**) comparison of calculated PDSI to Dai’s result; (**c**) spatial trend of PDSI during 1965–2014; (**d**) spatial trend of PDSI during 2000–2014. (Generated by ArcGIS 10.2, URL: http://www.esri.com/software/arcgis/arcgis-for-desktop, and Matlab 2012a, URL: http://cn.mathworks.com/products/matlab/).

To describe the overall statistics of recent changes in droughts over Central Asia, Fig. 1a plots the time series of the PDSI (mean ± error) and Fig. 1b compared our calculated PDSI to Dai’s result^1, 2^. As shown in Fig. 1b, the PDSI_Calcu. is a good match with PDSI_Dai. Furthermore, we find that the PDSI we calculated has, for the most part, remained at a normal level, showing a near-zero trend (0.0036 per year). Despite the smaller overall trend in PDSI, there was a switch since the early 2000s to a drying trend over the past decade (−0.023 per year) (Fig. 1a), generally following the dramatic increase in air temperature.

The spatial distribution of the PDSI in 1965–2014 shows a mixture of drying (42% of land area) and wetting (58% of land area) that combined to yield a slightly wetting trend. Northern Xinjiang in China, the Tienshan Mountains in Central Asia, and the western areas of Kazakhstan show somewhat increased (wetting) trends (Fig. 1c). Over the past decade (2000–2014), the spatial distribution of PDSI trends show drying across much of Central Asia (65% of the land area), whereas PDSI in western and eastern Kazakhstan, Turkmenistan, and Kyrghyzstan as well as the Tarim River basin in China reveal obvious declining (drying) trends (Fig. 1d).

### Quantifying the sensitivity of PDSI droughts

**Figure 2:**
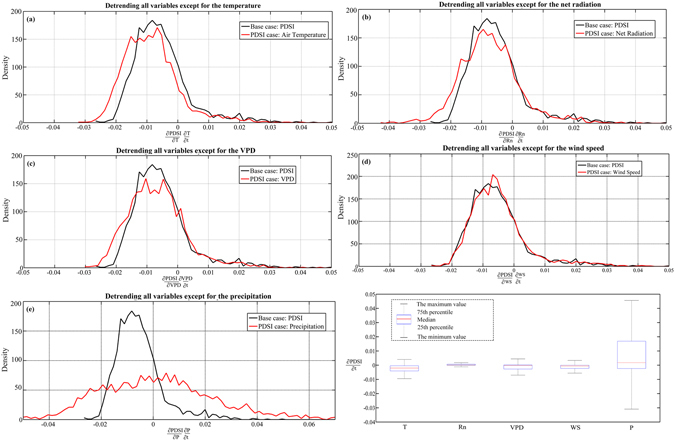
Quantifying the sensitivity of the changes in droughts over Central Asia using a series of numerical experiments. (**a**) The temperature case (detrending all variables except for the temperature): the probability density function (PDF) of the trends in the PDSI; (**b**) the net radiation case; (**c**) the vapour pressure deficiency case, (**d**) the wind speed case; (**e**) the precipitation case; (**f**) the summary of the contributions from different meteorological variables to the PDSI droughts. (Generated by Matlab 2012a, URL: http://cn.mathworks.com/products/matlab/).

A physical-based approach was used to quantify the sensitivity of PDSI droughts. We summarized the probability density function (PDF) of the PDSI trends, as shown in the detailed maps in Fig. 2. The widely used detrending approach eliminated the trend component from the observed climatic time series to form a new “detrended” time series. For the base case (black line) in Fig. 2, all meteorological variables were detrended for 1965–2014. Figure 2a is the temperature case, indicating that all variables were detrended except for air temperature. In this instance, the PDF of the PDSI moved to the left (dry) by −0.0025, which showed the response level of the PDSI drought to warming. The net radiation (Rn) (Fig. 2b) and vapor pressure deficiency (VPD) (Fig. 2c), were also causing a slight drying in the PDSI. The results showed that the response level of the PDSI drought to air temperature was higher than that to the other meteorological variables.

As it turns out, the PDF of the PDSI is broadened when the trend in precipitation is included, which is slightly wetting (moved to the right by 0.0018) but very small compared with the large variation (Fig. 2e). In contrast to the trend in the above-mentioned meteorological variables, the PDSI indicated a slightly positive (wetting) trend by releasing the observed trend in the wind speed (Fig. 2d). In fact, based on the summary of the contributions from several different meteorological variables to the PDSI droughts (Fig. 2f), the average changes in drought were very small compared with large spatial variations in the PDSI trend, which mainly arise from the spatial variations in the precipitation trend.

### Meteorological drought: fluctuant precipitation change

**Figure 3:**
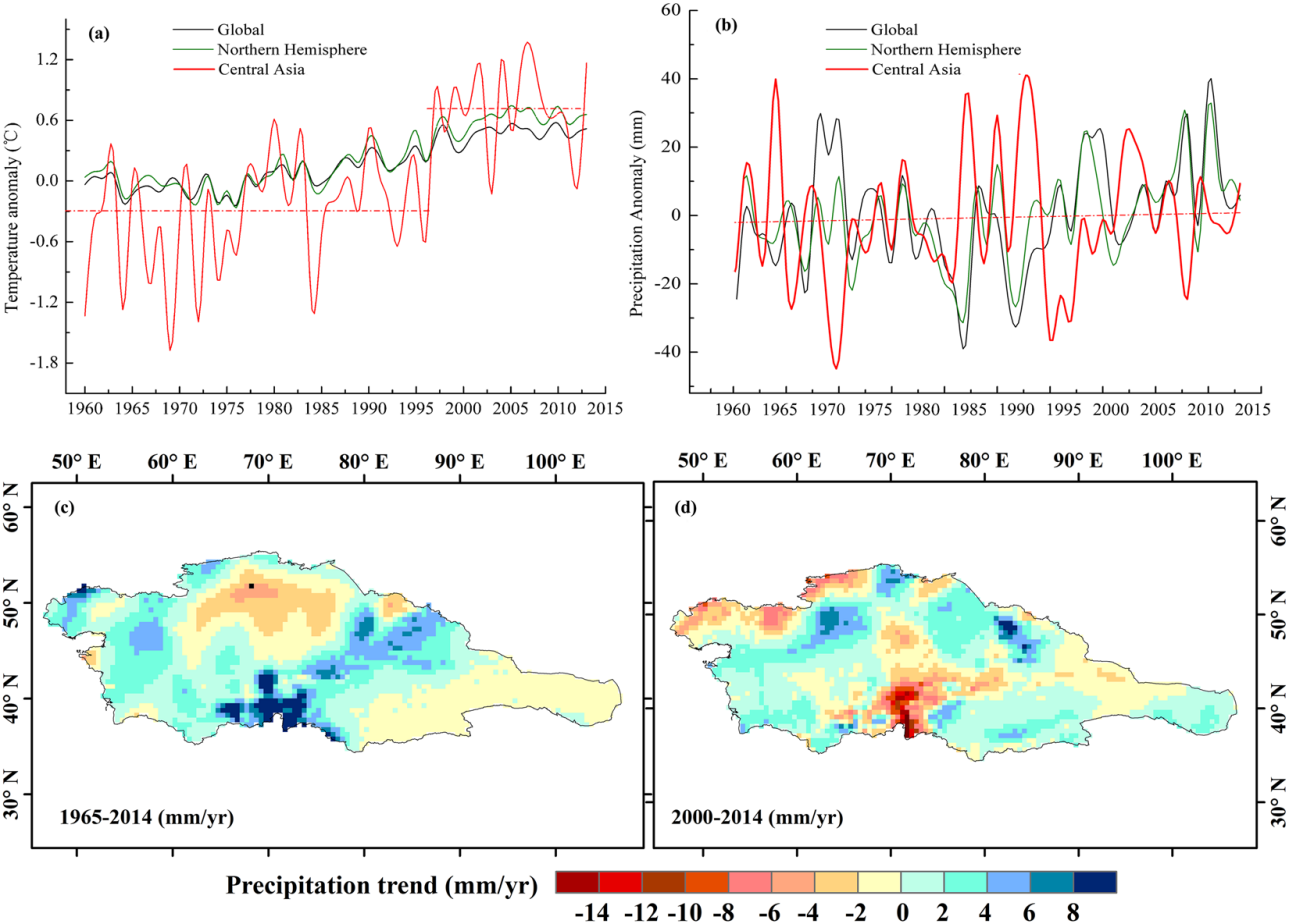
Spatio-temporal changes of temperature and precipitation during 1965–2014. (**a**) Annual temperature of Global, Northern Hemisphere and Central Asia; (**b**) annual precipitation of Global, Northern Hemisphere and Central Asia; (**c**) spatial trend of precipitation in Central Asia during 1965–2014; (**d**) spatial trend of precipitation in Central Asia during 2000–2014. (Generated by ArcGIS 10.2, URL: http://www.esri.com/software/arcgis/arcgis-for-desktop).

In examining regional precipitation anomalies under global warming, we found that precipitation in Central Asia remained relatively stable in 1965–2014 (Fig. 3b) and that decadal fluctuation amplitude was greater in Central Asia than the overall level globally or in the Northern Hemisphere. The 1990s were the most humid decade in the past half-century in Central Asia, but unlike the continuously increasing precipitation trends globally and in the Northern Hemisphere, precipitation in Central Asia showed a slightly downward trend from 2000 to 2014. The spatial distribution of precipitation trends in 1965–2014 show that the most significantly reduced area was northern Kazakhstan and the Hexi Corridor (Fig. 3c).

The average annual temperature in Central Asia experienced a significant increasing trend from 1965 to 2014, with each decade warmer than the previous ones, and experienced a sharp increase in the recent decade (Fig. 3a). In 2000–2014, which was the warmest decade on record, the spatial trend of precipitation experienced some notable changes. For instance, the precipitation trend decreased in northwestern Kazakhstan and Kyrgyzstan and especially in Tajikistan (Fig. 3d). Both spatial and temporal changes of the meteorological drought were almost consistent with PDSI changes. Based on the simple water balance model, the implicit assumption in that interpretation was that the total evapotranspiration (E) increased substantially faster than precipitation (P), leading to a more arid environment with less runoff (Q).

### Agro-ecological drought: enhanced soil moisture drying

Available soil moisture is defined as the amount of water a plant can access in its root zone. The spatial and temporal variations in soil moisture are closely related to the ecosystem^30, 31^. Soil moisture is an important sensor for measuring surficial wetness and dryness levels, and closely reflects the dryness and wetness of climate. The concurrent drought and heatwave events with low soil moisture and high actual evapotranspiration (ET_a_) triggered the agro-ecological drought.

**Figure 4:**
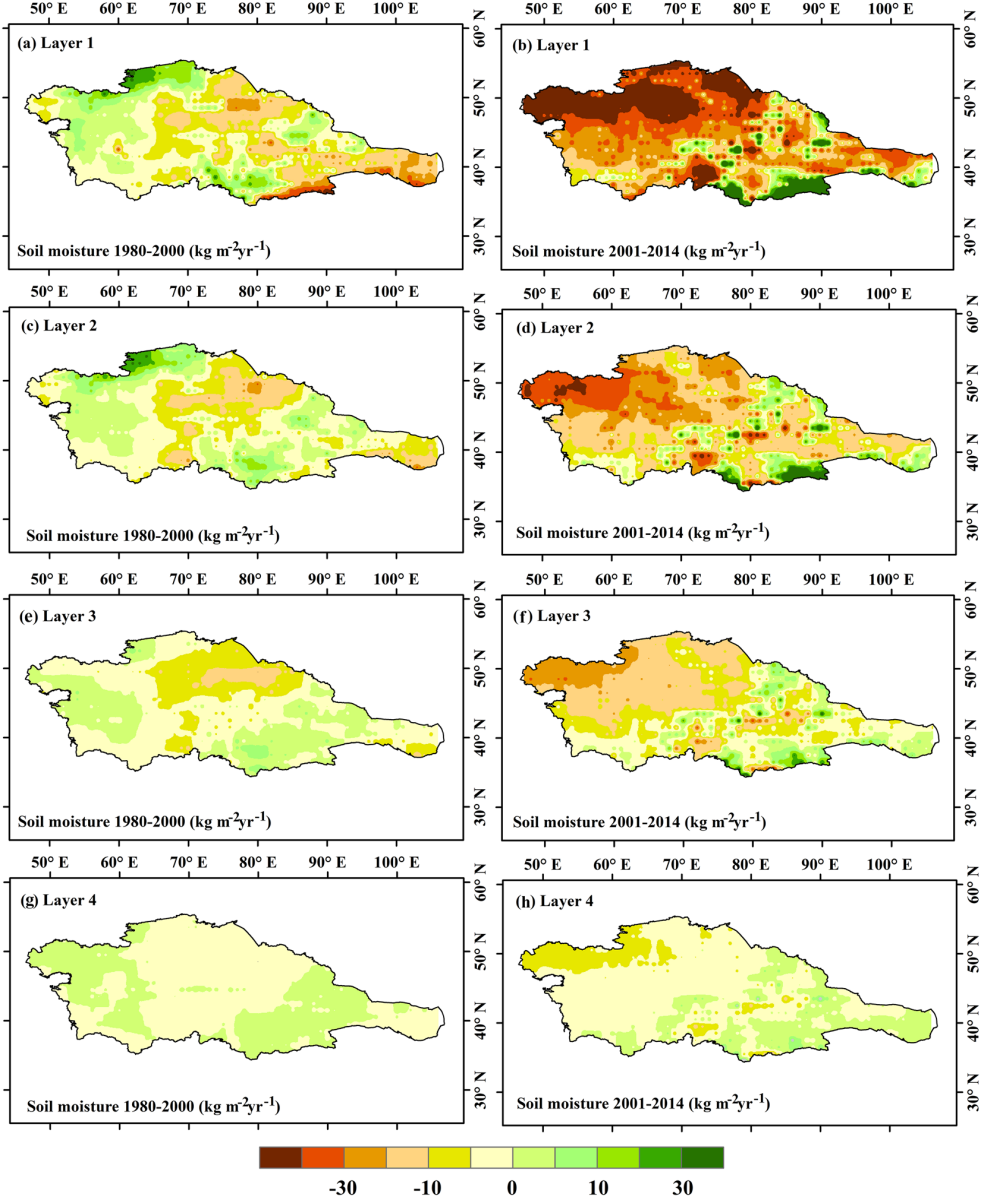
Average soil moisture in four layers during 1980–2014. (Generated by ArcGIS 10.2, URL: http://www.esri.com/software/arcgis/arcgis-for-desktop).

Soil moisture has exhibited a long-term decreasing trend over the past several decades, and this is particularly obvious in the most recent (and warmest) decade. The spatial distribution of annual soil moisture trends illustrates a total decrease in the soil moisture of four specific layers (0–10, 10–40, 40–100, and 100–200 cm) in Central Asia, especially of the shallow soil layers (Fig. 4). Prior to 2000, 65% of these areas were negative and 35% were positive in layer 1 (Fig. 4a), 63% were negative and 37% were positive in layer 2 (Fig. 4c), 68% were negative and 32% were positive in layer 3 (Fig. 4e), and 61% were negative and 39% were positive in layer 4 (Fig. 4g). The most prominent drying trends occurred in 1980–2000 in the middle section of Central Asia (i.e., eastern Kazakhstan, Kyrgyzstan and Tajikistan). In the recent warmest decade (2001–2014), the soil moisture trend was comparable with various PDSI readings. About 84% were negative and 16% were positive in layer 1 (Fig. 4b), 81% were negative and 19% were positive in layer 2 (Fig. 4d), 79% were negative and 21% were positive in layer 3 (Fig. 4f), and 72% were negative and 28% were positive in layer 4 (Fig. 4h). The most prominent drying trends occurred in Kazakhstan in 2001–2014, especially in northwestern Kazakhstan.

**Figure 5:**
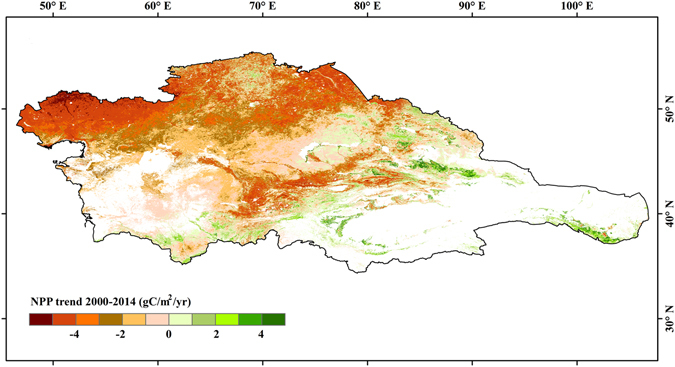
Spatial pattern of NPP trend during 2000–2014. (Generated by ArcGIS 10.2, URL: http://www.esri.com/software/arcgis/arcgis-for-desktop).

Vegetation in Central Asia is generally characterized as desert vegetation mixed with mountain and oasis vegetation patches. Plants with this type of growth mainly depend on shallow soil water and shallow groundwater to survive. Hence, the soil moisture drying process of the past two decades has intensified desertification and also led to water-based ecosystems becoming significantly more fragile in arid regions. For instance, the NDVI of natural vegetation in Central Asia exhibited an increasing trend at a rate of 0.004 per decade prior to 1998, after which the trends reversed and the NDVI decreased at a rate of 0.003 per decade^26^. Terrestrial net primary production (NPP) can be defined as the amount of photosynthetically-fixed carbon available to the first heterotrophic level^32–34^. Plant productivity in temperate ecosystems was subject to soil moisture depletion from increased transpiration^35^. Broadly comparable to soil moisture variations in the recent decade, the annual NPP decreased significantly in Central Asia, particularly in northwestern Kazakhstan and some parts of Kyrgyzstan and Tajikistan (Fig. 5). In the arid region of northwest China (e.g., northern slope of the Tienshan Mountains, southern margin of the Tarim Basin, and the Hexi Corridor), NPP experienced an increasing trend. The same regions also had increased soil moisture.

### Rising ET_a_ exacerbates regional ecological droughts in the past decade

ET_a_ was a crucial variable that amplified the soil moisture loss^36^ and had contributions from three main components: direct evaporation from the soil (E_s_), transpiration from vegetation (E_c_), and vaporization of intercepted rainfall from vegetation (E_i_). The relative contributions from E_s_ and E_i_ were larger in Central Asia. E_s_ was mostly driven by the atmospheric demand for vapour, the availability of water in the soil, and the amount of vegetation above the soil, while E_c_ was dependent on plant phenology and water-use efficiency, and E_i_ by the occurrence of rainfall and the characteristics of the vegetation stand^37^.

**Figure 6:**
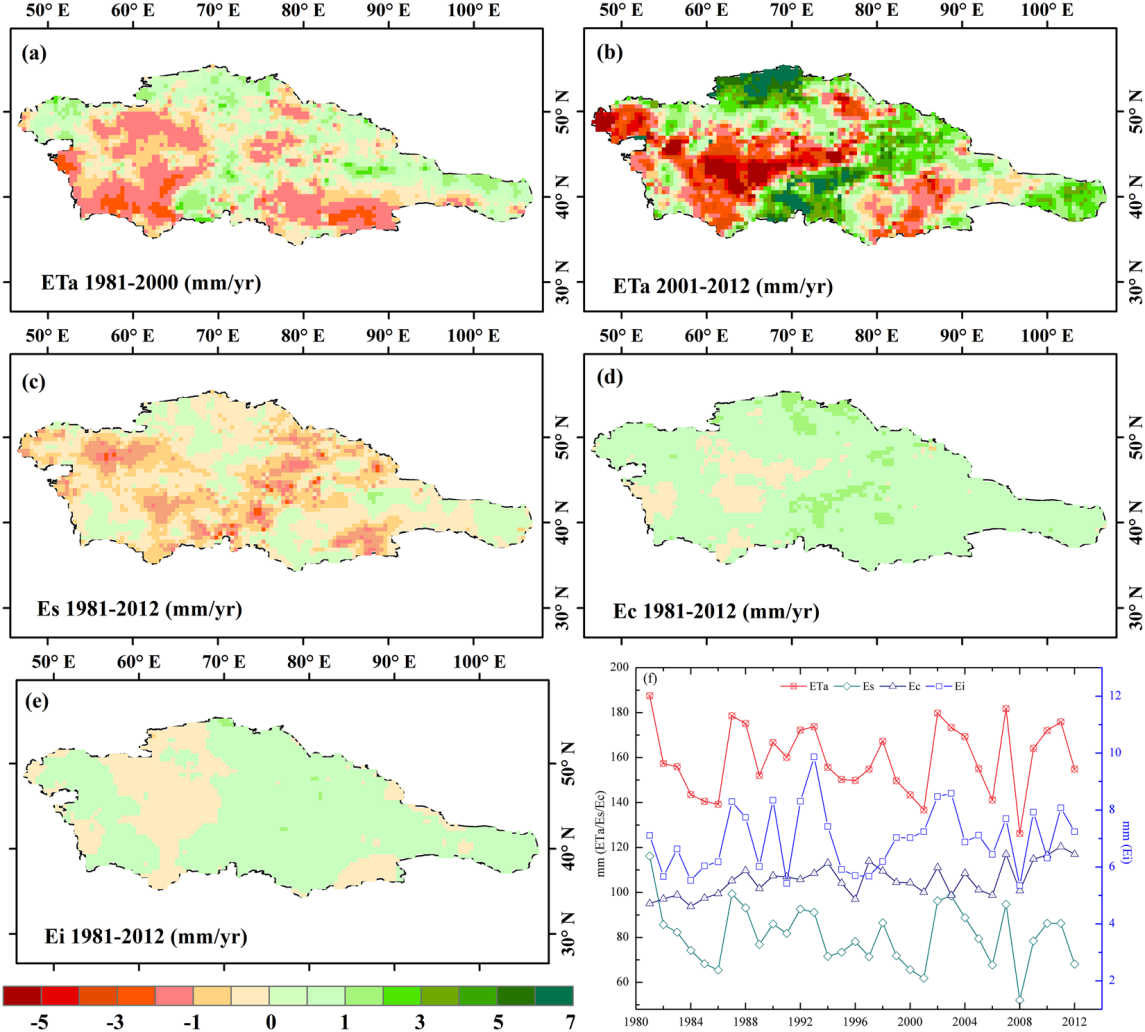
Trend differences of ET_a_ and its components. (**a**) ET_a_ in 1981–2000; (**b**) ET_a_ in 2001–2012; (**c**) E_s_ in 1981–2012; (**d**) E_c_ in 1981–2012; (**e**) E_i_ in 1981–2012; (**f**) time series of ET_a_, E_s_, E_c_ and E_i_. (Generated by ArcGIS 10.2, URL: http://www.esri.com/software/arcgis/arcgis-for-desktop, and Matlab 2012a, URL: http://cn.mathworks.com/products/matlab/).

In areas characterized by less moisture, increases in precipitation were insufficient to offset increases in evaporative demand as land-surface temperatures rose^38^. The multi-decadal declining trend (1981–2012) in ET_a_ continued until about 2000, and then gradually turned slightly positive. The average inter-annual variance of ET_a_ was −0.42 mm/yr in 1981–2000, while inter-annual variance of ET_a_ was 0.29 mm/yr since 2000 (Fig. 6f). The increased areas of ET_a_ were distributed in northern Kazakhstan, Kyrgyzstan and Turkmenistan, intensifying over the past decade (Fig. 6a and b). This was mainly the result of increases in E_c_. Negative ET_a_ trends were observed over parts of Uzbekistan and Turkmenistan and were mainly explained by reductions in E_s_ and E_i_ in these regions. Spatially, E_c_ and E_i_ had an overall rising trend at a rate of 0.45 mm/yr and 0.02 mm/yr, respectively, in 1981–2012 (Fig. 6d and e). The value and trend difference in E_i_ was much smaller compared to that in E_s_ and E_c_. Specifically, E_s_ decreased at a rate of −0.33 mm/yr (Fig. 6c), which partly counter-balanced the contribution of E_c_ and E_i_ to ET_a_. Shallow soil water was a more immediate buffer for E_s_ responding directly to the precipitation variability. Shallow soil moisture shortage could be the reason for the decline of E_s_. The contrasting positive trend in E_c_ and negative trend in E_s_ was mostly explained by the increase in the leaf area index^37^.

ET_a_ responds to variability in atmospheric conditions rather than variability in soil moisture. In the past decade, the increased potential (as a surrogate measure of atmospheric moisture demand) and actual evapotranspiration are accelerating the soil water consumption at low values. This, coupled with a significant decline in water storage and shallow groundwater levels^39^, is causing the shallow roots of desert plants to die. A consistent increase in evapotranspiration acts to exacerbate global water deficits and amplify the risk of ecological drought.

### Hydrological drought: different spatial and temporal distribution of runoff

**Figure 7:**
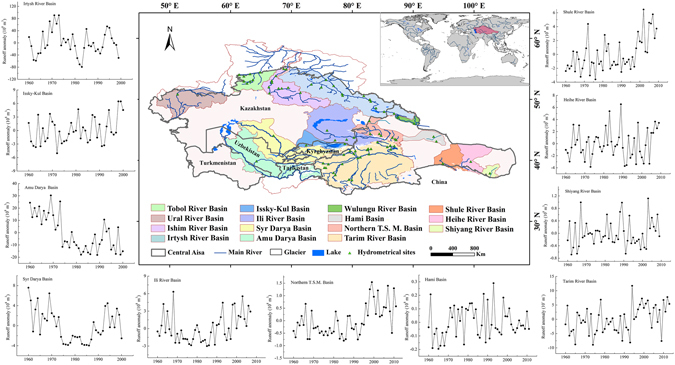
Distribution and runoff changes in the main 11 large rivers in Central Asia in the past half century. (Generated by ArcGIS 10.2, URL: http://www.esri.com/software/arcgis/arcgis-for-desktop, and Matlab 2012a, URL: http://cn.mathworks.com/products/matlab/).

Hydrological drought is often diagnosed as stream flow drought. We used long-term runoff observations from 11 main large headwater catchments in Central Asia that covered a wide range of hydroclimatological conditions. Variations in runoff in select rivers in Central Asia over the past half-century are shown in Fig. 7. As can be see, other than for the Amu Darya, Syr Darya and Hami basins (decreased trend), river runoffs have shown increasing trends since the 1960s, especially in the Northern Tienshan Mountain Basins and the Shule River Basin, where runoffs increased significantly (*p* = 0.01).

River runoff responds in a complex way to changes in climate and the cryosphere in arid and semi-arid regions^27^. The dry trend regions (PDSI) showed dry trends of runoff for four basins (Amu Darya Basin, Syr Darya Basins, Hami Basin, and Irtysh River Basin), and wet trend regions showed wet trends of runoff for two basins (Northern Tienshan Mountains Basin and Heihe River Basin). Meanwhile, dry trend regions showed wet trends of runoff for four basins (Ili River Basin, Issky-Kul Basin, Tarim River Basin and Shule River Basin), and a wet trend region showed a dry trend of runoff (Shiyang River Basin). Although these dry trend regions, catchments here with a higher fraction of glacierized areas showed mainly increasing runoff trends in the past, e.g., the Tarim River Basin (with a glacier area of 15830 km^2^) and the Ili River Basin (with a glacier area of 2111 km^2^). Hydrological droughts are not entirely comparable to PDSI, which may indicate that more frequent hydrological droughts are resulting not only from lower precipitation but also from other factors influencing water balance conditions in these basins. Meltwater from glacierized catchments is one of the most important water supplies in central Asia. Climate-driven changes in glacier/snow-fed runoff regimes have significant effects on water supplies^27^. It mainly related to runoff recharge proportions from glaciers and precipitation had great effects on the relation between runoff and temperature and precipitation in different rivers. Catchments with a higher fraction of glacierized area showed mainly increasing runoff trends although there with a declined precipitation. River Basins with less or no glacierization exhibited large variations in the observed runoff changes due to precipitation anomalies.

## Discussion

### Reassessment of drought indices’ type and approach

Several drought indices, such as the Standardized Precipitation Index (SPI)^9^, the standardized precipitation evapotranspiration index (SPEI)^40^ and the Reconnaissance Drought Index (RDI)^41^, are based on precipitation alone or precipitation and evaporation. Hence, they are useful as a measure of precipitation deficits or meteorological drought, but are limited to representing regional real droughts. The PDSI takes this one step further by accounting for the balance of precipitation, evapotranspiration and runoff, and has the ability to incorporate local soil and possibly also vegetation properties, making it a fairly comprehensive and flexible index of relative drought^17^.

However, there are apparently conflicting PDSI results when using two different ways to estimate the PET, i.e., the widely used Thornthwaite^12^ approach (available input data: monthly mean temperatures) and the Penman-Monteith^29^ approach (require more diverse input data). With the warming, the droughts become dramatically more severe in the PDSI__ Thornthwaite_ than in the PDSI__PM_. A detailed comparison of various forcing data for the PDSI calculations claimed that PDSI__PM_ gives a better estimate of the true trend in global drought because of its more comprehensive physics^8, 11^. Even so, both climatologies and changes in actual/potential evapotranspiration over time have been extremely difficult to determine reliably^42^, and the discrepancies between the previous studies are also likely due to the precipitation data sets used (e.g., the Climate Research Unit [CRU] has very recently updated their precipitation data set). We used contemporary data, which are the best currently available globally, but they are still not perfect.

### Lack of valuable and comprehensive discussion of regional real droughts

As mentioned above, while the method for estimating PDSI using a physical-based approach has now been brought closer to the key water balance components, there still remains a lack of comprehensive discussion around regional real droughts. Fundamentally, agro-ecological and hydrological drought relates to the amount of water available in soils or hydrological systems.

Soil temperature and soil water content explained 70–80% of the seasonal variability of ecosystem respiration. During the drought, light-saturated ecosystem gross carbon uptake decreased during the drought^43^. Once vegetation suffers persistent drought, the vegetation biomass will rapidly decline and further intensify the drought. If low levels of soil moisture persist for long enough, reductions in natural vegetation cover and vigor can occur. Otherwise, the feedbacks of vegetation to the drought are complicated in the short-term and long-term period in different regions^36, 38^. For future variation of regional drought risk, it is necessary to consider the mutual feedback of agro-ecological drought and ecosystem.

Zhai *et al*.^9^ calculated drought indices (PDSI and SPI) from 483 meteorological stations in China from 1961–2005, explained the correlation between runoff anomalies and the annual average PDSI and SPI for ten large regions covering the territory of China, and concluded that about six large rivers (runoff in these rivers are generated primarily from precipitation) had significant correlations. Runoff trends indicate a complex response of catchments to climate changes in water-limited regions. Our results showed less than adequate co-variability between the PDSI and observed runoff. With further warming and the subsequent acceleration of glacier retreat, glacier inflection points will or have already appeared. The amount of surface water will probably decline or maintain high volatility due to glacial retreat and the reduced storage capacity of glaciers. As the climate changes, recent hydrological data may be a poor guide to future drought, and planned actions may prove insufficient to maintain adequate water supplies^16^. Runoff trends indicate a complex response to changes in climate. For future variation of hydrological drought, it is essential to quantify the responses of hydrologic processes to both climate change and shrinking glaciers/snow in glacierized catchments. Therefore, using the drought index (relative measures) to evaluate droughts (absolute terms) might not be the best approach.

### Expectations for changes in global drought

Sheffield *et al*.^10^ claimed there was little change in global drought over the past 60 years, with a mixture of increases (58% of land area) and decreases (42%) for the PDSI__PM_, and most of the drying occurring across northern North America, central South America, Africa, eastern Russia, southeast Asia, and eastern Australia. Recent analyses of model-simulated soil moisture^22^, drought indices^4, 25^ and precipitation-minus-evaporation^24^, however, do suggest increased risk of drought in the twenty-first century. The patterns are characterized by drying over most of Africa, southeast Asia, eastern Australia and southern Europe, and increased wetness over the central US, Argentina, and northern high-latitude areas. The basis for the previous interpretation that aridity will increase with future CO_2_-induced warming was that the projected increase in potential evaporation was substantially larger than the projected increase in precipitation over land. Models predict that India and northern tropical Africa will become wetter, but nearly all other land regions are predicted to become drier in the next century^44^. How can the data provide such seemingly contradictory conclusions? There are obvious reasons to be cautious in making conclusions about how drought is changing globally. Reconciling these differences is necessary before the model predictions can be trusted, and the input data sets employed remain a key concern.

### Attribution of droughts

Some regions have suggested that higher temperatures than normal were the cause for increased drought in recent years through increased evaporation. Drought and heat go together and both have increased from human and natural causes^19, 45^. Flash droughts averaged over China increased by 109% from 1979 to 2010, followed by contributions from decreasing soil moisture and increasing ET; these droughts are most likely to occur over humid and semi-humid regions^46^. Yet there is evidence that the direct impact of temperature on drying may actually be a misinterpretation of feedback between the land and the atmosphere. Regional changes in near-surface humidity, soil moisture, and precipitation are tightly coupled^44^. In some regions, evaporation actually decreases during drought because of less precipitation. Yin *et al*.^21^ interpreted that the decline in precipitation during drought reduces the available water, thereby decreasing evapotranspiration.

In a water-limited environment, there is minimal runoff, and any reduction in precipitation during a meteorological drought will generally result in a reduction in soil moisture. Dai^25^ concluded that the warming that has occurred since the 1980s (the jump is due to the 1982/1983 El Niño) has contributed considerably to the upward trend in global drought areas, increasing the areas under drought by about 8% by the first decade of the present century. The warming-induced drying results from increased evaporation and is most prevalent over northern mid-high latitudes. In contrast, decreases in precipitation in Africa, southeast Asia, eastern Australia and southern Europe are the primary cause for the drying trend in those regions.

The atmospheric circulation creates favourable conditions for drought, which can be initiated by sea surface temperature anomalies. The most common source of episodic droughts around the world is the El Niño/Southern Oscillation (ENSO). During El Niño events, there are major droughts over Australia, Indonesia, southeast Asia, parts of Africa and northeastern Brazil^17^. Thus, as ENSO is the primary source of variability in the tropical and global precipitation record, variations in ENSO affect perceptions about changes in drought^47^.

Cheng and Huang^22^ concluded that, beginning in the 1980s, noticeable drying first took place in humid transitional regions and extended to dry transitional regions. For the global average, precipitation had a dominant effect on the variability of soil moisture at inter-annual to decadal time scales, but temperature was the main cause of the long-term trend of soil moisture. This is consistent with the hypothesis that plant productivity in temperate ecosystems is subject to soil moisture limitations during summer^48^. They also suggesting that a positive solar radiation anomaly could either increase the photosynthetic response reflected by a positive NDVI anomaly, or in some instances cause soil moisture depletion from increased transpiration with the opposite effect^35^.

All of the above-mentioned expectations are based on correlation analysis, as quantitative attribution to drought is difficult. To this end, we used a detrending technique^49^ to make all climatic forcings form a new climatic time series, and then designed a base case to quantify the sensitivity of drought to climatic variables based on the PDSI__PM_ approach.

As the above considerations show, focusing on changes in drought indices does not indicate a regional real drought, and perhaps does not even represent the main hydrological drought in drylands basins. Instead, a comprehensive assessment for measuring long-term drought that recognizes meteorological, hydrological and agro-ecological is needed. Furthermore, focusing on changes in temperature and precipitation does not provide comprehensive attributions, especially in severe droughts that occurred in human-dominated environments^19^. The increasing occurrence of drought, enhanced warming, and rapidly growing human population will exacerbate the risk of land degradation and desertification, especially in the drylands of developing countries.

## Methods

### Penman-Monteith method in estimating the PDSI

Potential evapotranspiration (PET) is one of the inputs in the Palmer Drought Severity Index (PDSI). PET estimates based on the Penman-Monteith approach^29^ are considered to be more physically realistic but require more diverse input data.

PDSI drought is estimated by the difference between the observed monthly precipitation and required “precipitation” that is estimated under climatically appropriate for existing conditions (CAFEC) for each month^50^.

1 $$\mathop{p}\limits^{\frown {}}={\mathop{E}\limits^{\frown {}}}_{T}+\mathop{R}\limits^{\frown {}}+{\mathop{R}\limits^{\frown {}}}_{O}-\mathop{L}\limits^{\frown {}}$$

where ${\mathop{E}\limits^{\frown {}}}_{T}$ = actual evapotranspiration, $\mathop{R}\limits^{\frown {}}$ = moisture stored in soil system, ${\mathop{R}\limits^{\frown {}}}_{O}$ = runoff, and $\mathop{L}\limits^{\frown {}}$ = moisture loss in soil system

2 $${\mathop{E}\limits^{\frown {}}}_{T}=\alpha PET,\alpha =\frac{\overline{{E}_{T}}}{\overline{PET}}$$

3 $$\mathop{R}\limits^{\frown {}}=\beta PR,\beta =\frac{\bar{R}}{\overline{PR}}$$

4 $${\mathop{R}\limits^{\frown {}}}_{O}=\gamma P{R}_{O},\gamma =\frac{{\bar{R}}_{O}}{\overline{P{R}_{O}}}$$

5 $$\mathop{L}\limits^{\frown {}}=\delta PL,\delta =\frac{\bar{L}}{\overline{PL}}$$

where $\overline{PET}$ = average potential evapotranspiration, $\overline{PR}$ = average recharge, $\overline{PR0}$ = average runoff, and $\overline{PL}$ = average moisture loss.

This research adopts the Penman-Monteith method to estimate daily mean potential evapotranspiration (mm d^−1^).

6 $$PET=\frac{0.408{\rm{\Delta }}({R}_{{\rm{n}}}-G)+\gamma \frac{900}{{T}_{{\rm{mean}}}+{\rm{273}}}{U}_{2}({{\rm{vp}}}_{{\rm{s}}}-{\rm{vp}})}{{\rm{\Delta }}+\gamma ({\rm{1}}+{\rm{0}}{{\rm{.34U}}}_{2})}$$

where PET is potential evapotranspiration, R_n_ is the net radiation at the crop surface (MJ m^−2^ d^−1^), G is the soil heat flux density (MJ m^−2^ d^−1^), T_mean_ is the air temperature at 2 m height (°C), u_2_ is the wind speed at 2 m height (m s^−1^), vp_s_ is the saturation vapor pressure (kPa), vp is the actual vapor pressure (kPa), Δ is the slope vapor pressure curve (kPa °C^−1^), and γ is the psychrometric constant (kPa °C^−1^). Here we transformed the observed wind speed data at 10 metres above the ground to 2 metres.

Available moisture stored in soil system (*PR*) and available runoff (*PR*_*0*_):

7 $$PR=AWC-({S}_{s}+{S}_{u})$$

8 $$P{R}_{0}=AWC-PR=({S}_{s}+{S}_{u})$$

where *S*_*s*_ = available moisture stored in the surface layer at the start of the month, *S*_*u*_ = available moisture stored in underlying levels at the start of the month, and AWC = available capacity of the soil system.

Moisture loss in soil system (*PL*):

9 $$PL=PLs+P{L}_{u}$$

10 $$PLs=\,{\rm{\min }}(PET+{S}_{s})$$

11 $$P{L}_{u}=\frac{(PET-P{L}_{s}){S}_{u}}{AWC},P{L}_{u}\le {S}_{u}$$

where *PL*_*s*_ = moisture loss from surface layer and *PL*_*u*_ = moisture loss from underlying layer.

Difference between the actual precipitation and the CAFEC precipitation (*d*):

12 $$d=P-\mathop{P}\limits^{\frown {}}$$

The monthly moisture anomaly index (*Z*) is defined as:

13 $$Z={K}_{j}{d}_{j}$$

where *K*_*j*_ = monthly constants.

The monthly Palmer Drought Severity Index is defined as:

14 $$PDS{I}_{i}=0.897PDS{I}_{i-1}\frac{1}{3}{Z}_{i}$$

### Quantifying the sensitivity of PDSI drought

Keeping one variable and detrending the rest may affect the interdependencies of the meteorological variables^11^. We applied a detrending technique^49^ to make all climatic forcings (meteorological variables) form a new climatic time series, and then designed a base case to examine the PDSI when all climatic forcings are free of any trends. For example, one meteorological factor (only) case is the numerical experiment where all other meteorological variables are detrended. We designed five numerical experiments based on the variables of PDSI and P-M approaches, e.g., the temperature case: detrending all variables except for the temperature.

15 $${T}_{detrend,i}={T}_{observed,i}+\alpha (i-1965)$$

where *T*_*detrend,i*_ is the detrended temperature in year *i* and *T*_*observed,i*_ is the original (observed) temperature in the same year, *i* denotes the year 1965, 1966, …, 2014.

For other climatic variables, we first eliminated the trend component in climatic annual time series:

16 $${F}_{y-{\rm{\det }}rend,i}={F}_{y-observed,i}+\alpha (i-1965)$$

We then applied the annual ratio to the daily timescale to the daily timescale to generate a daily timeseries:

17 $${F}_{detrend,i}={F}_{observed,i}(\frac{{F}_{y-detrend,i}}{{F}_{y-observed,i}})$$

### Trend analysis

We examined linear trends estimation on a per-pixel basis to establish a linear regression relationship between variables (x_i_) and time (t_i_). The regression coefficient (b) is:

18 $${\rm{b}}=\frac{n\times \sum _{i=1}^{n}xiti-\sum _{i=1}^{n}{x}_{i}\,\sum _{i=1}^{n}ti}{n\times \sum _{i=1}^{n}t{i}^{2}-{(\sum _{i=1}^{n}ti)}^{2}}$$

### Data analysis

The monthly grid data of the temperature and precipitation series (1965–2014), with a spatial resolution of 0.5 degree, were collected from the Climatic Research Unit (http://www.cru.uea.ac.uk/data/) developed by the Climatic Research Unit (University of East Anglia) in conjunction with the Hadley Centre (at the UK Met Office).

To calculate the PET, which was used to calculate the PDSI_Calcu., we collected the data of air temperature, maximum temperature, minimum temperature, pressure, relative humidity, U-wind, V-wind, net shortwave radiation and net longwave radiation from the NCEP/NCAR Reanalysis 1 (http://www.esrl.noaa.gov/psd/data/gridded/data.ncep. reanalysis.html)^51^. We unified the spatio-temporal resolution of these data from different sources based on re-sampling and re-classification techniques. As an indicator of land-surface moisture conditions, PDSI has been widely used for the routine monitoring and assessment of global and regional drought conditions.

Global monthly 0.5 degree spatial resolution actual evapotranspiration and its components (transpiration, soil evaporation, interception) from 1981 to 2012 were collected from CSIRO (http://doi.org/10.4225/08/5719A5C48DB85). The estimates were computed through the observation-driven Penman-Monteith-Leuning (PML) model. This data compared the estimated mean annual ET_a_ (1981–2012, mm year^−1^) to catchment ET_a_ (P–Q) observations in 643 catchments, which showed good correspondence^37^.

The soil moisture data comes from the Global Land Data Assimilation System (http://disc.sci.gsfc.nasa.gov/uui/datasets?keywords=Hydrology), with the spatial resolution of 0.25 degree. The depths of the four soil layers are: 0–10 cm, 10–40 cm, 40–100 cm, and 100–200 cm. The quality of the GLDAS data set was assessed against available observations from multiple sources^52, 53^.

Monthly surface runoff data from 1960–2012 (e.g., Ili River Basin, Northern Tienshan Mountains Basin, Hami Basin, Tarim River Basin, Shule River Basin, Heihe River Basin and Shiyang River Basin) were collected at the flow controlling sites out of the mountains in each river from the Regional Hydrological Bureau in China. Monthly surface runoff data in rivers outside of China (Syr Darya Basin, Amu Darya Basin, Issky-Kul Basin and Irtysh River Basin) were collected from the Global Runoff Data Centre and relevant references. It is worth noting that the hydrological monitoring stations and routine surveillance programs have ceased to operate following the breakdown of the Soviet Union.
